# Metallothionein II treatment mitigates rotenone-induced neurodegeneration in zebrafish models of Parkinson’s disease

**DOI:** 10.3389/fphar.2025.1478013

**Published:** 2025-01-31

**Authors:** Yong Hui Nies, Wei Ling Lim, Norwahidah Abd Karim, Mohamad Fairuz Yahaya, Seong Lin Teoh

**Affiliations:** ^1^ Department of Anatomy, Faculty of Medicine, Universiti Kebangsaan Malaysia, Kuala Lumpur, Malaysia; ^2^ Department of Biological Sciences, School of Medical and Life Sciences, Sunway University, Petaling Jaya, Malaysia; ^3^ Department of Biochemistry, Faculty of Medicine, Universiti Kebangsaan Malaysia, Kuala Lumpur, Malaysia

**Keywords:** neurodegenerative disease, neuroprotection, pesticide, dopaminergic neuron, mitochondrial function

## Abstract

**Introduction:**

Parkinson’s disease (PD) is a common neurodegenerative disorder primarily affecting motor function due to progressive loss of dopaminergic neurons in the substantia nigra. Current therapies offer symptomatic relief but fail to halt disease progression, highlighting the need for novel therapeutic strategies. This study explores the neuroprotective potential of exogenous human metallothionein 2 (hMT2) peptide in a rotenone-induced PD zebrafish model.

**Methods:**

Adult zebrafish were divided into four groups: control, rotenone-treated, hMT2 pre-treatment, and hMT2 co-treatment. PD model was established by exposing zebrafish to 5 µg/L rotenone water for 28 days. hMT2 (0.2 µg) was administered intracranially either one day before or seven days after rotenone exposure.

**Results:**

The novel tank test demonstrated that rotenone exposure significantly impaired locomotor activity (*p* < 0.05) and increased anxiety-like behavior (*p* < 0.001). Additionally, PD model zebrafish exhibited reduced dopamine levels, decreased dopaminergic neuron population, elevated oxidative stress, heightened inflammatory response and mitochondrial dysfunction. Treatment with hMT2, especially in the co-treatment group, ameliorated these deficits by restoring locomotor activity, dopamine levels, and dopaminergic neuron counts while reducing oxidative stress and inflammation, and improving mitochondrial function.

**Discussion:**

These results suggest that hMT2 exhibited neuroprotective effect in the PD model zebrafish. These findings support the potential of MT as a therapeutic agent for PD.

## Introduction

Parkinson’s disease (PD) is the second most common neurodegenerative disease after Alzheimer’s disease (Poewe et al., 2017). PD primarily affects individuals over the age of 65, with a rapid increase in prevalence among those older than 80 (Ou et al., 2021). PD patients experience various motor impairments, including rigidity, resting tremor, bradykinesia, postural instability and speech difficulties (Hor et al., 2020; Chan et al., 2021). These motor symptoms result from the progressive degeneration of dopaminergic neurons in the substantia nigra pars compacta, along with the accumulation of intracellular inclusions containing α-synuclein aggregates, known as Lewy bodies (Masato et al., 2019). In addition to motor symptoms, PD patients often experience numerous non-motor symptoms, such as autonomic dysfunctions (gastrointestinal, urogenital, cardia, respiratory), neuropsychiatric dysfunctions (cognitive, mood, dementia), olfactory loss and sleep disorders (insomnia, daytime sleepiness, or rapid eye movement) (Peball et al., 2020). These non-motor symptoms often precede the onset of motor symptoms and significantly affect the quality of life in PD patients (Azmin et al., 2014). Hospitalization is often required to manage these symptoms, with PD-related admissions linked to greater disease severity, leading to more complications and extended hospital stays, compared to non-PD-related admissions (Shaibdat et al., 2023).

Despite extensive research, the pathogenesis of PD remains poorly understood. Aging, genetic susceptibility and environmental factors are believed to interact in ways that contribute to dopaminergic neuronal loss (Nies et al., 2021). Exposure to environmental toxins such as metals and pesticides is a significant risk factor for PD (Chambers-Richards et al., 2023). Rotenone, a pesticide that inhibits mitochondrial complex I, thereby impairing mitochondrial function, has been positively associated with PD, and is widely used to model the disease in animal and *in vitro* studies (Tanner et al., 2011; Najib et al., 2020).

Although the exact cause of PD has not been identified, various treatments ranging from drugs to surgeries and therapies have been introduced to manage PD symptoms. However, none of these treatments prevent or slow the disease progression (Lee and Yankee, 2021), highlighting the need for more effective therapies.

Metallothioneins (MT) are small, cysteine-rich proteins composed of a single polypeptide chain of 61–68 amino acids, with four major isoforms identified in mammals, namely, MT1, -2, -3 and -4 (Merlos Rodrigo et al., 2020). While in zebrafish, 2 MT isoforms have been identified: *mt2* and *similar to mtb* (*smtb*). Both genes are expressed in cell-proliferating regions in the adult zebrafish brain, such as the ventricular layers in the telencephalon, diencephalon, mesencephalon and rhombencephalon, suggesting their roles in neurogenesis and neuroprotection (Teoh et al., 2015). MTs are known to regulate essential metal homeostasis (primarily for zinc and copper), detoxify heavy metal, regulate zinc-metalloprotein biosynthesis and activity, and protect cells from free radical-induced damage (Teoh, 2016). Previous study has reported an increase in the expression of MT (*MT1E*, *MT1F*, *MT1G*, *MT1H*, *MT1M*, *MT1X* and *MT2A*) in the substantia nigra and frontal cortex of PD patients (Michael et al., 2011). Given their important biological roles, MTs have been explored as potential therapeutic targets to counteract copper-induced α-synuclein aggregation (Okita et al., 2017) and oxidative stress (Ooi et al., 2020). This study aims to investigate the protective effects of exogenous full-length human MT2 peptide (hMT2) in a rotenone-induced PD zebrafish model.

## Methodology

### Animals and experimental design

Adult zebrafish (*Danio rerio*, 4-months old, equal numbers of male and female, n = 72), purchased from a local aquarium supplier, were housed in 6 L tanks at 27°C ± 0.5°C with a controlled light-dark cycle (14 h light and 10 h dark). The fish were fed commercially available food (New Life Spectrum, Thera + A, United States) twice daily.

**FIGURE 1:**
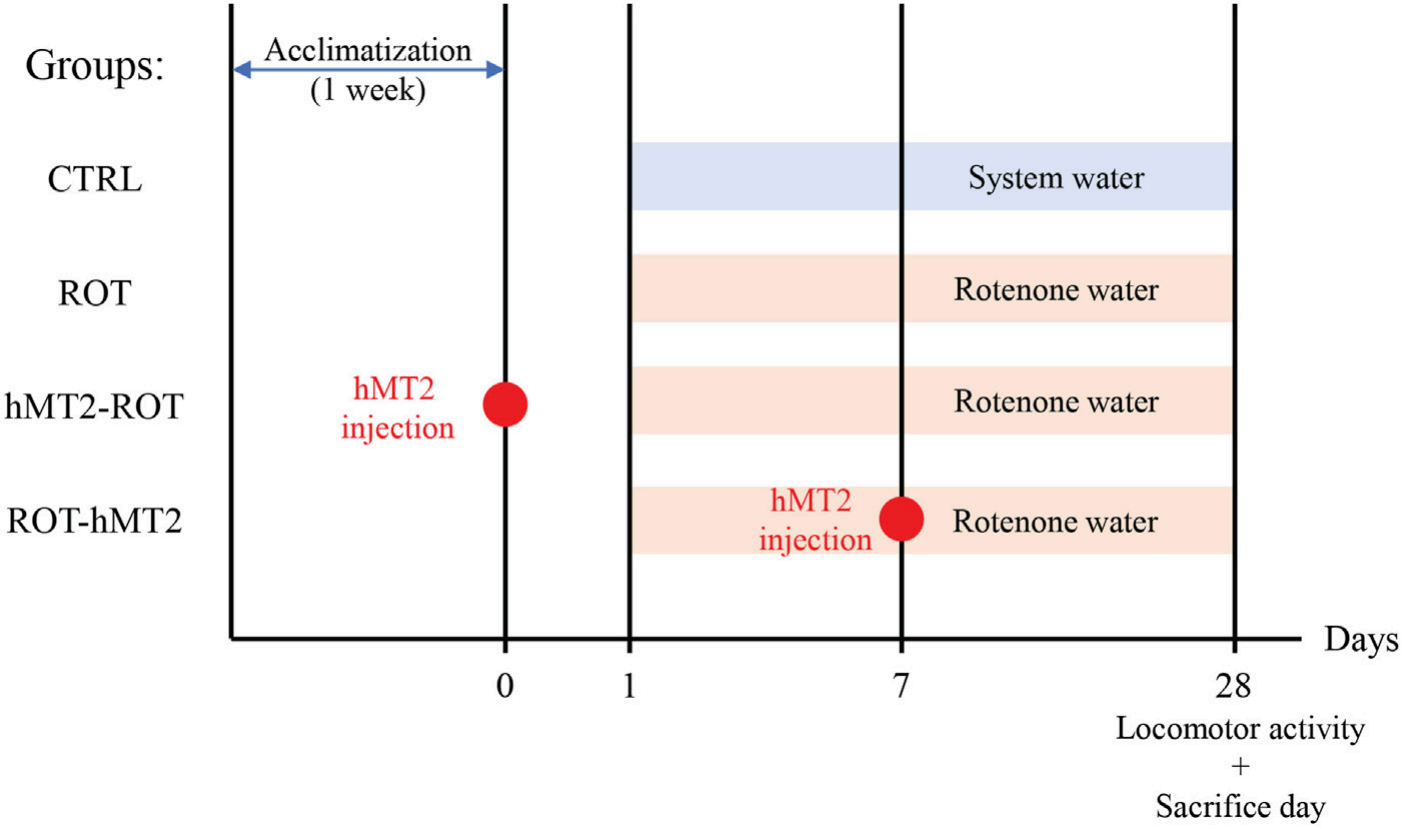
Experimental design of animal groups and treatment timeline. Fish were divided into 4 groups (n = 15 per group), namely, control (CTRL), rotenone-treated PD model (ROT), hMT2 pretreatment (hMT2-ROT), and hMT2 co-treatment (ROT-hMT2) groups. PD was induced in zebrafish using 5 μg/L rotenone water for 28 days. hMT2 was intracranially injected a day before (hMT2-ROT) or 7 days after (ROT-hMT2) the rotenone exposure.

Fish were randomly divided into 4 groups (n = 15 per group), namely, control (CTRL), rotenone-treated PD model (ROT), hMT2 pretreatment (hMT2-ROT), and hMT2 co-treatment (ROT-hMT2) groups (Figure 1). Following a 1-week acclimatization period, all experimental procedures were conducted in accordance with the ethical guidelines approved by the Animal Ethics Committee of Universiti Kebangsaan Malaysia (Approval no: ANAT/FP/2020/SEONG LIN/16-JAN./1076-JAN.-2020-DEC.-2020).

### Rotenone and hMT2 treatment

All preparation and handling of rotenone (Cat#8875, Sigma, St Louis, MO, United States) were conducted under a fume hood and decontaminated with 1% bleach water. For PD model induction, zebrafish were exposed to 5 μg/L rotenone (Cat#R8875, Sigma, United States) in water for 28 days, with fresh rotenone water renewed every 48 h (Khotimah et al., 2015).

Fish were anesthetized with 0.0035% benzocaine (Sigma, United States) prior to intracranial hMT2 (Cat#AB112324, Abcam, Cambridge, England) injection. Each fish received a 0.2 μg dose of hMT2, administered intracranially either 1 day before (hMT2-ROT) or 7 days after (ROT-hMT2) rotenone exposure. The selected dose of 0.2 µg/fish was based on a prior study that demonstrated neuroprotective effects of hMT2 in MPTP-induced zebrafish PD models (Mohamad Najib et al., 2023). A syringe (BD PrecisionGlide 30G × 0.5′, BD Medical, Franklin Lakes, NJ, United States) was used to create an entry point in the skull without injuring the brain. The hMT2 solution was then injected into the cranial cavity through this opening using a heat-pulled glass microcapillary pipette attached to a microinjector (IM-9B, Narishige, Tokyo, Japan).

### Zebrafish locomotor analysis–novel tank test

Fish underwent the novel tank test after 28 days of rotenone exposure. Locomotor analysis was performed according to a modified protocol by Teoh (2016). Briefly, each fish was placed in a transparent acrylic tank (242 mm L × 99 mm W × 100 mm H), containing 2.5 L of dechlorinated system water. The fish swimming behaviour was recorded from a side-view using a webcam (C920 HD Pro Webcam, Logitech, Lausanne, Switzerland), positioned approximately 0.5 m away from the tank, for 5 min. The recorded videos wers analysed using digital video tracking software (SMART 3.0.02, Panlab Harvard Apparatus, Holliston, MA, United States).

### Quantitative PCR analysis

Fish were sacrificed after 28 days of exposure to system water (CTRL) or rotenone water (ROT, hMT2-ROT, ROT-hMT2) by immersion in iced-cold water. Whole brains were dissected under a stereomicroscope, and total RNA was extracted using TRIzol reagent (Invitrogen, Carlsbad, CA, United States) according to the manufacturer’s protocol. First-strand cDNA was synthesized from 500 ng of the total RNA using ProtoScript II First Strand cDNA Synthesis Kit (Cat #E6560L, New England BioLabs, Ipswich, MA, United States) in a 20 µL reaction mixture, according to the manufacturer’s protocol.

qPCR was performed on the cDNA using CFX96 Touch Real-Time PCR Detection System (Bio-Rad Laboratories, Hercules, CA, United States) to evaluate the expression of zebrafish MT (*mt2* and *smtb*), dopamine transporter (*dat*, involved in dopamine uptake), tyrosine hydroxylase (TH) (*th1* and *th2*, involved in dopamine biosynthesis), proinflammatory cytokines (interleukin (*il*)*-1a*, *-1b*, cyclooxygenase-2 (*cox2*) and tumor necrosis factor alpha (*tnfa*), and brain-derived neurotrophic factor (*bdnf*). All gene-specific primers used in this study are summarized in Table 1. *β-actin* mRNA expression was used for normalization. Each 20 μL reaction mixture contained 10 µL Luna Universal qPCR Mix (Cat #M3003L, New England BioLabs, United States), 10 μM forward and reverse primers each and 1 μL of sample cDNA. The non-template control was applied with distilled water. The qPCR cycling conditions were 95°C for 10 min, followed by 40 cycles of 95°C for 15 s and 60°C for 1 min. Relative gene expression was analyzed using the 2-ΔΔCq method.

**TABLE 1:** Gene specific primers for qPCR.

Gene	Abbreviation	Accession number	qPCR primer sequence (5′ → 3′)
Metallothionien 2	*mt2*	NM_001131053.2	F:TGCGAATGTGCCAAGACTGG
			R:CACTTGCAGGTAGCACCACA
Similar to metallothionein-B	*smtb*	NM_001201469.1	F:GACCAGTGTGACTGCTCCAA
			R:TGCCGCAACTTTTCTTGTCG
Dopamine transporter	*dat*	AF318177.1	F: GAG​TCG​GGT​TTG​GTG​TGC​TA
			R: GGC​GTC​TCT​GTA​GCA​GTT​GT
Tyrosine hydroxylase 1	*th1*	NM_131149.1	F: GAA​CAT​GGC​GGG​AGG​TCT​AC
			R: TCC​AGT​AAC​CGG​AAA​GCC​TC
Tyrosine hydroxylase 2	*th2*	NM_001001829.1	F: TTA​GCG​TTC​CGG​GTT​TTC​CA
			R: CAG​CAA​TCT​GGT​TCA​GGG​GA
Interleukin 1α	*il1a*	NM_001290418.1	F:AGCACCATGTCAGCACCAAT
			R:GGAACACCTGGATTGTCCACT
Interleukin 1β	*il1b*	NM_212844.2	F:TTTGTGGGAGACAGACGGTG
			R:TGTAGCTCATTGCAAGCGGA
Cyclooxygenase-2	*cox2*	NM_153657.1	F:GTCATGGAGTGGATCTGGGG
			R:TGTTGAACCTCCAGCGTCTC
Tumour necrosis factor α	*tnfa*	NM_212859.2	F:CTCCATAAGACCCAGGGCAA
			R:TGGCAGCCTTGGAAGTGAAA
Brain-derived neurotrophic factor	*bdnf*	NM_131595.2	F:AGTTGCGCGGAGGTCTTATC
			R:GTTGGAACTTTACTGTCCAGTCG
Beta actin	*β-actin*	NM_131031.2	F: CAC​GCT​CAG​CAT​TGT​GAG​TT
			R: CAA​CCA​TCA​CTC​CCT​GAT​GTC

### Measurement of brain dopamine levels

Dissected whole brains (n = 6 per group) were homogenized in phosphate-buffered saline (PBS). Dopamine levels were measured using a Fish dopamine ELISA kit (Cat#SL0046FI, Sunlong Biotech, Hangzhou, China), following the manufacturer’s protocol.

### Assessment of brain lipid peroxidation

Lipid peroxidation in brain tissue samples was measured using Quantichrom™ TBARS Assay Kit (BioAssay Systems, Hayward, CA, United States) according to the manufacturer’s protocol. Briefly, 20 mg of brain tissue was homogenized in 200 μL of cold PBS, followed by the addition of 200 μL of 10% trichloroacetic acid. Samples were then transferred to a 96 well plate. The optical density (OD) was measured at 535 nm (range 525–545 nm) with a monochromator microplate reader (Multiskan™ FC Microplate Photometer, Thermo Fisher Scientific, Waltham, MA, United States). Results were expressed as nmol MDA/mg protein.

### Dopaminergic neuron immunohistochemistry and image analysis

Whole brains (n = 3 per group) were fixed in 4% paraformaldehyde/phosphate buffered fixative for 6 h, and cryoprotected in 20% sucrose solution overnight at 4°C before embedded in Tissue-Tek optimal cutting temperature compound (Sakura Finetechnical, Tokyo, Japan). Coronal brain sections (15 μm thickness) were cut with a cryostat and thaw-mounted onto 3-aminopropylsilane (APS)-coated glass slides (Muto Pure Chemicals, Tokyo, Japan).

Sections were incubated with mouse anti-rat TH antibody (diluted 1:500, Cat#22941, Immunostar, Hudson, NY, United States) and Animal Research Kit ARK™ Peroxidase (Cat#K3954, Agilent, Santa Clara, CA, United States) in a humid chamber for 24 h at 4°C. Color development was carried out using 0.05% 3,3′diaminobenzidine tetrahydrochloride (DAB) with 0.03% hydrogen peroxide (H_2_O_2_) in 0.05M Tris-HCl (pH 7.5). Sections were cleared in xylene following dehydration, cover-slipped and sealed with DPX mountant (Fisher Chemical, New Jersey, United States). Photomicrographs were captured with the Pannoramic MIDI scanning system (3DHISTECH, Budapest, Hungary), and processed with the CaseViewer Image Software 2.4 (3DHISTECH, Hungary). TH immunoreactive (TH^+^) cells were quantified from individual sections of 15 μm, located approximately 60 μm apart within each brain, from at least 3 sections per brain and 3 brains per group.

### Mitochondria function assay

Mitochondria were isolated from zebrafish brain tissues using mitochondria isolation kit (Cat#AB110169, Abcam, Cambridge, England), and stored at −80°C before further analysis. Mitochondrial function were assessed using the MitoPlate™ I-1 (Biolog, Hayward, CA, United States) following the manufacturer’s instructions. Briefly, isoloted mitochondria were added into wells of the MitoPlate I-1, and OD readings at 590 nm were recorded continuous for 6 h at 5 min intervals with a monochromator microplate reader.

### Statistical analysis

All data were expressed as mean ± SEM. Statistical analysis was conducted using the Statistical Package for the Social Sciences version 26 (IBM, Armonk, New York, United States). Analysis was carried out using independent t-test and/or ANOVA followed by *post hoc* Tukey’s test, and *p* < 0.05 was considered significant.

## Results

### Effects of rotenone and hMT2 on locomotor activity

**FIGURE 2:**
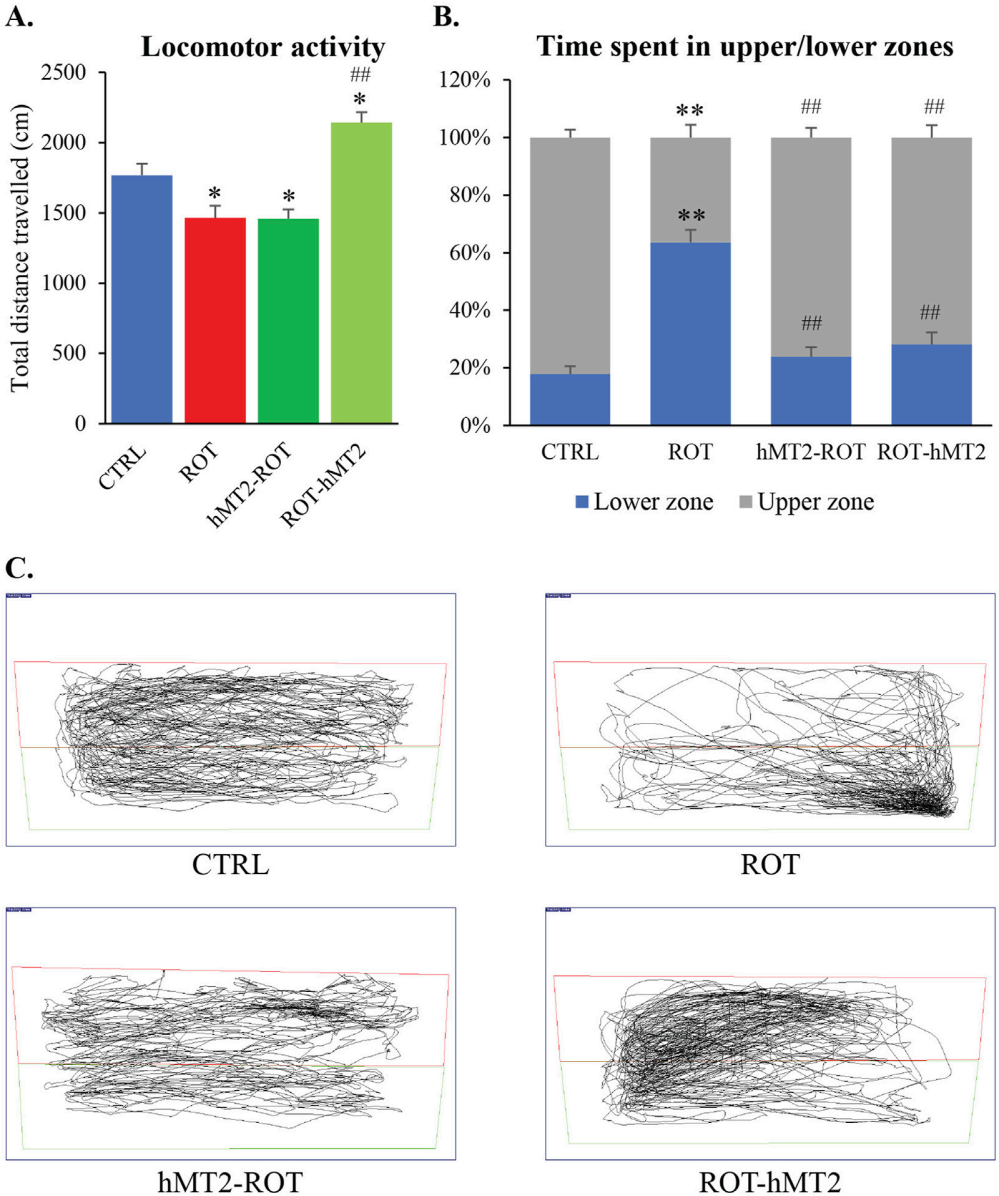
Effects of rotenone on locomotor activity of adult zebrafish. **(A)** Locomotor analysis measured the total distance traveled for 5 min in a novel tank. **(B)** Total time of the experimental fish explored the upper and lower zones of the tank. **(C)** Representative computerized video tracking of zebrafish’s path in the novel tank over 5 min. *p < 0.05, **p < 0.001 vs. CTRL; #p < 0.05, ##p < 0.001 vs. ROT (n = 6 per group).

Locomotor activity of zebrafish was assessed after 28 days of exposure to 5 μg/L rotenone to evaluate the potential ameliorating effects of hMT2 on motor dysfunction in this PD model. The ROT group demonstrated a significant reduction in total swimming distance compared to the CTRL group (*p* < 0.05, Figure 2A). In contrast, the ROT-hMT2 group showed a significant increase in total swimming distance compared to the ROT group (p < 0.001). However, the hMT2-ROT group did not show a significant difference in locomotor activity compared to ROT group.

The novel tank test was also used to quantify anxiety-like behavioral responses based on the vertical distribution of fish. The ROT group spent a significantly longer swimming time in the lower zone of the tank compared with the CTRL group, indicating heightened anxiety levels (p < 0.001, Figure 2B). In contrast, CTRL fish spend more time exploring the upper zone of the tank. Notably, both the hMT2-ROT and ROT-hMT2 groups spent significantly more time in the upper zone compared to the ROT group (p < 0.001, Figure 2C), suggesting reduced anxiety.

### Effects of rotenone and hMT2 on *metallothioniein* and *bdnf* gene expression

**FIGURE 3:**
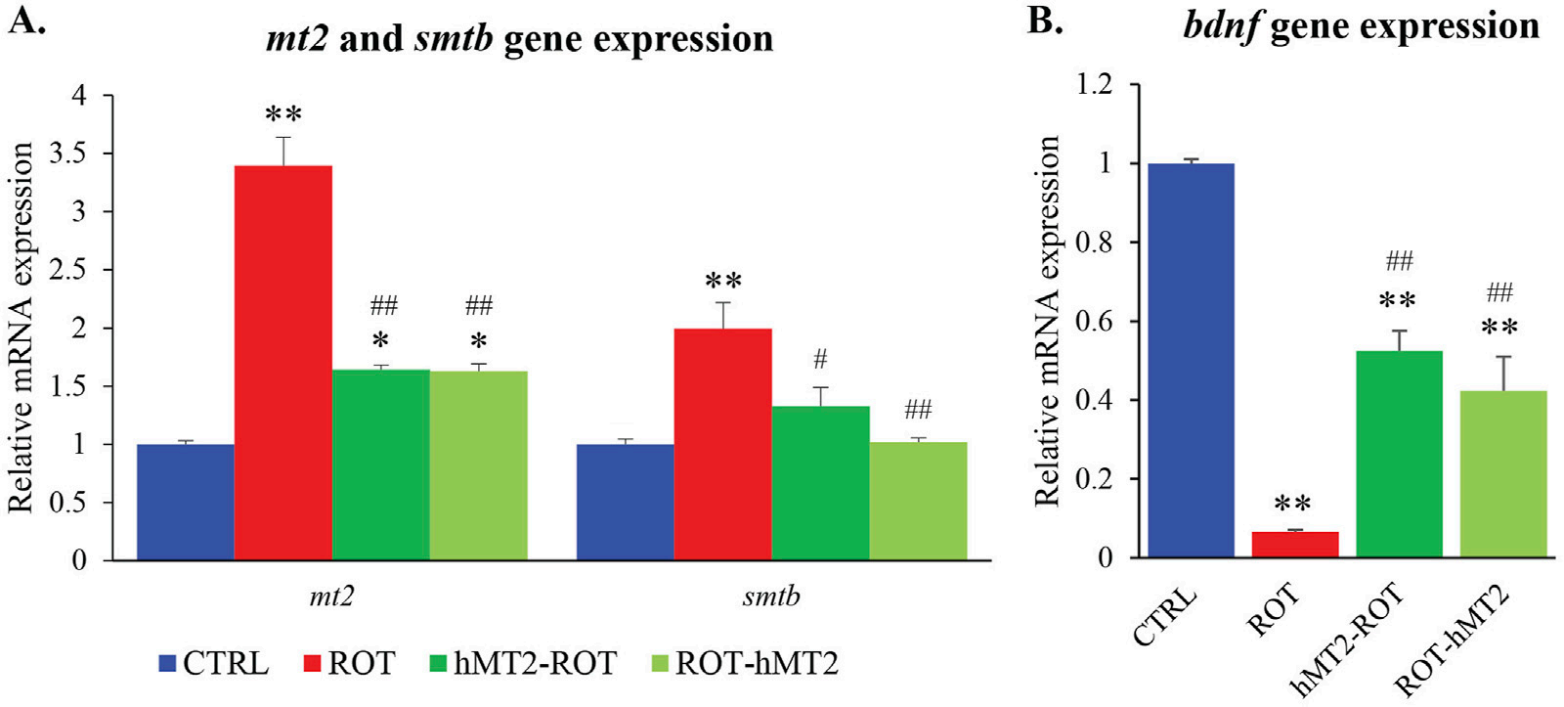
Effects of rotenone and hMT2 on metallothioniein and *bdnf* gene expression. Bar graph presentation of the fold change of **(A)** *mt2* and *smtb*, and **(B)** *bdnf* quantified by qPCR. All qPCR results are normalized to reference gene *β-actin*, and expressed as changes from the respective CTRL group. *p < 0.05, **p < 0.001 vs. CTRL; #p < 0.05, ##p < 0.001 vs. ROT (n = 6 per group).

The ROT group showed a significant upregulation of *mt2* and *smtb* gene expression compared to the CTRL group (p < 0.001, Figure 3A). In contrast, both the hMT2-ROT and ROT-hMT2 groups showed a significant downregulation in *mt2* and *smtb* gene expression compared to the ROT group (p < 0.05). Additionally, *bdnf* expression was significantly downregulated in the ROT group compared with the CTRL group (p < 0.001, Figure 3B). However, both the hMT2-ROT and ROT-hMT2 groups showed a singnificant upregulation in *bdnf* expression (p < 0.001) compared with the ROT group.

### Effects of rotenone and hMT2 on dopamine-related genes expression and dopamine levels

**FIGURE 4:**
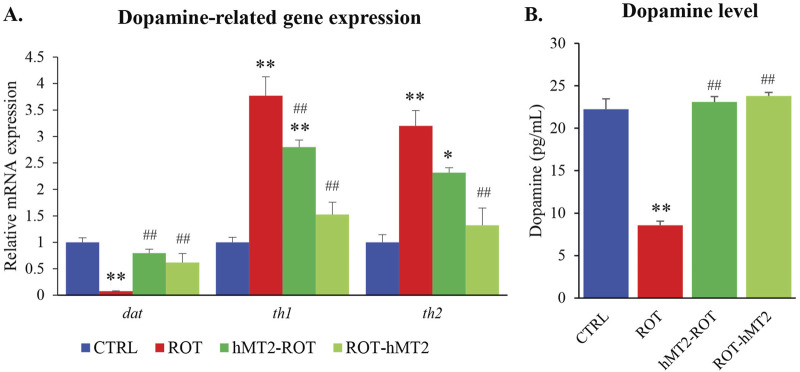
Effects of rotenone and hMT2 on dopamine-related genes expression and dopamine levels. **(A)** Bar graph presentation of the fold change of *dat*, *th1* and *th2* quantified by qPCR. All qPCR results are normalized to reference gene *β-actin*, and expressed as changes from the respective CTRL group. **(B)** Dopamine levels (pg/mL) of the groups. *p < 0.05, **p < 0.001 vs. CTRL; #p < 0.05, ##p < 0.001 vs. ROT (n = 6 per group).

In the ROT group, *dat* gene expression was significantly downregulated compared to CTRL group (p < 0.001, Figure 4A). The reduction was effectively restored to control levels with hMT2 treatments (p < 0.05). Additionally, *th1* and *th2* gene expression was significantly elevated in the ROT group compared to the CTRL group (p < 0.001). Both the hMT2-ROT (p < 0.05) and ROT-hMT2 (p < 0.001) treatment groups showed a significant downregulation in *th1* and *th2* gene expression compared to the ROT group.

Dopamine levels in the brain were also significantly reduced in the ROT group compared to the CTRL group (p < 0.001, Figure 4B). Treatment with hMT2 in both the hMT2-ROT and ROT-hMT2 effectively mitigated the rotenone-induced dopamine depletion in the zebrafish brain.

### Effects of rotenone and hMT2 on brain inflammation (pro-inflammatory cytokine expression and lipid peroxidation)

**FIGURE 5:**
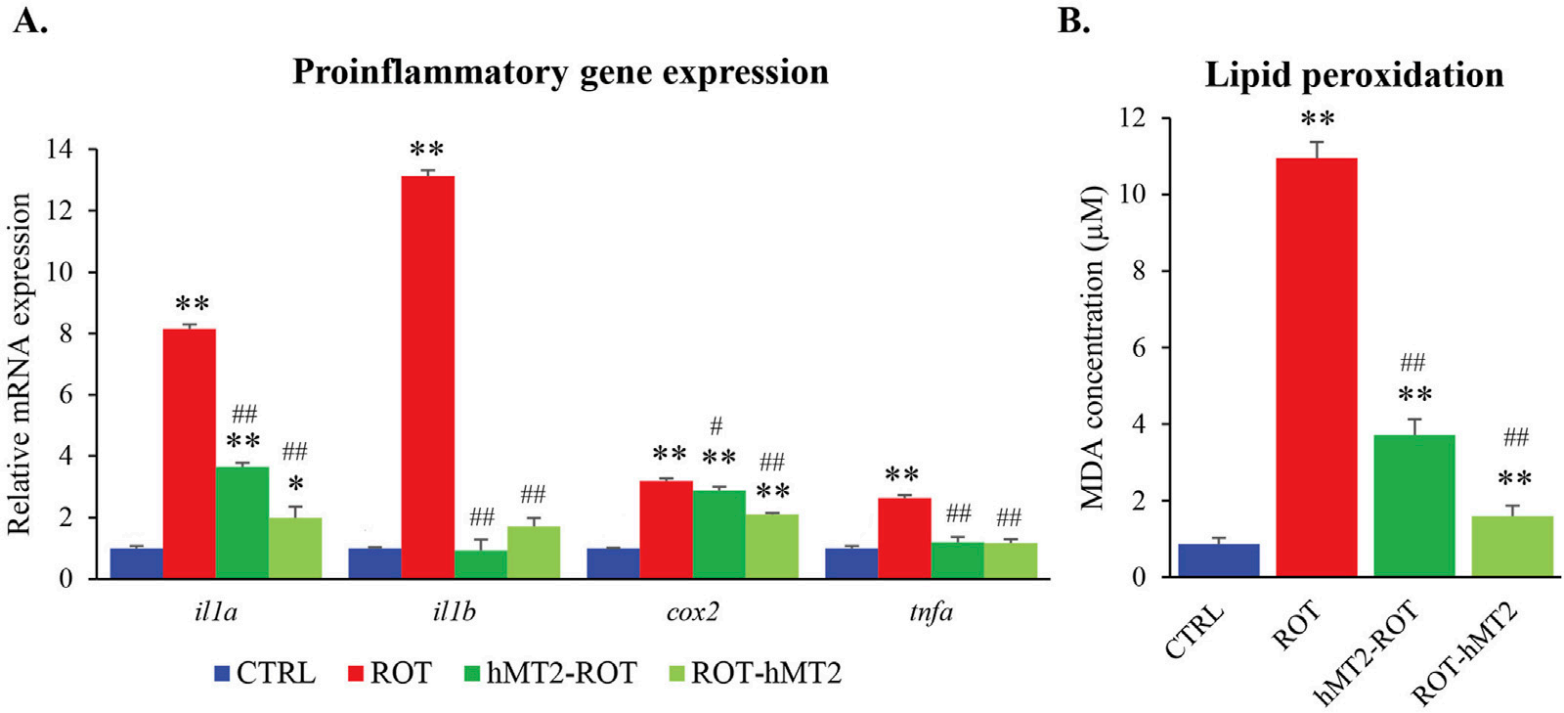
Effects of rotenone and hMT2 on brain inflammation. **(A)** Bar graph presentation of the fold change of *il1a*, *il1b*, *cox2* and *tnfa* quantified by qPCR. All qPCR results are normalized to reference gene *β-actin*, and expressed as changes from the respective CTRL group. **(B)** Lipid peroxidation (MDA, µM) of the groups. *p < 0.05, **p < 0.001 vs. CTRL; #p < 0.05, ##p < 0.001 vs. ROT (n = 6 per group).

The expressions of proinflammatory cytokines (*il1a*, *il1b*, *tnfa* and *cox2*) were significantly upregulated in the ROT group compared to the CTRL group (p < 0.001, Figure 5A). Both hMT2 treatment groups demonstrated a significant decrease in *il1a*, *il1b* and *tnfa* gene expression, restoring these cytokines to levels comparable to those of the control. Additionally, the ROT-hMT2 group showed a moderate but significant decrease in *cox-2* expression compared to the ROT group (p < 0.001).

Lipid peroxidation in zebrafish brain was assessed by TBARS production. The ROT group exhibited a significant increase in TBARS levels compared to the CTRL group (p < 0.001, Figure 5B). Treatment with hMT2 significantly reduced TBARS levels in both the hMT2-ROT and ROT-hMT2 groups compared to the ROT group, with decreases of 66% and 85%, respectively (p < 0.001).

### Effects of rotenone and hMT2 on dopaminergic neuron population

**FIGURE 6:**
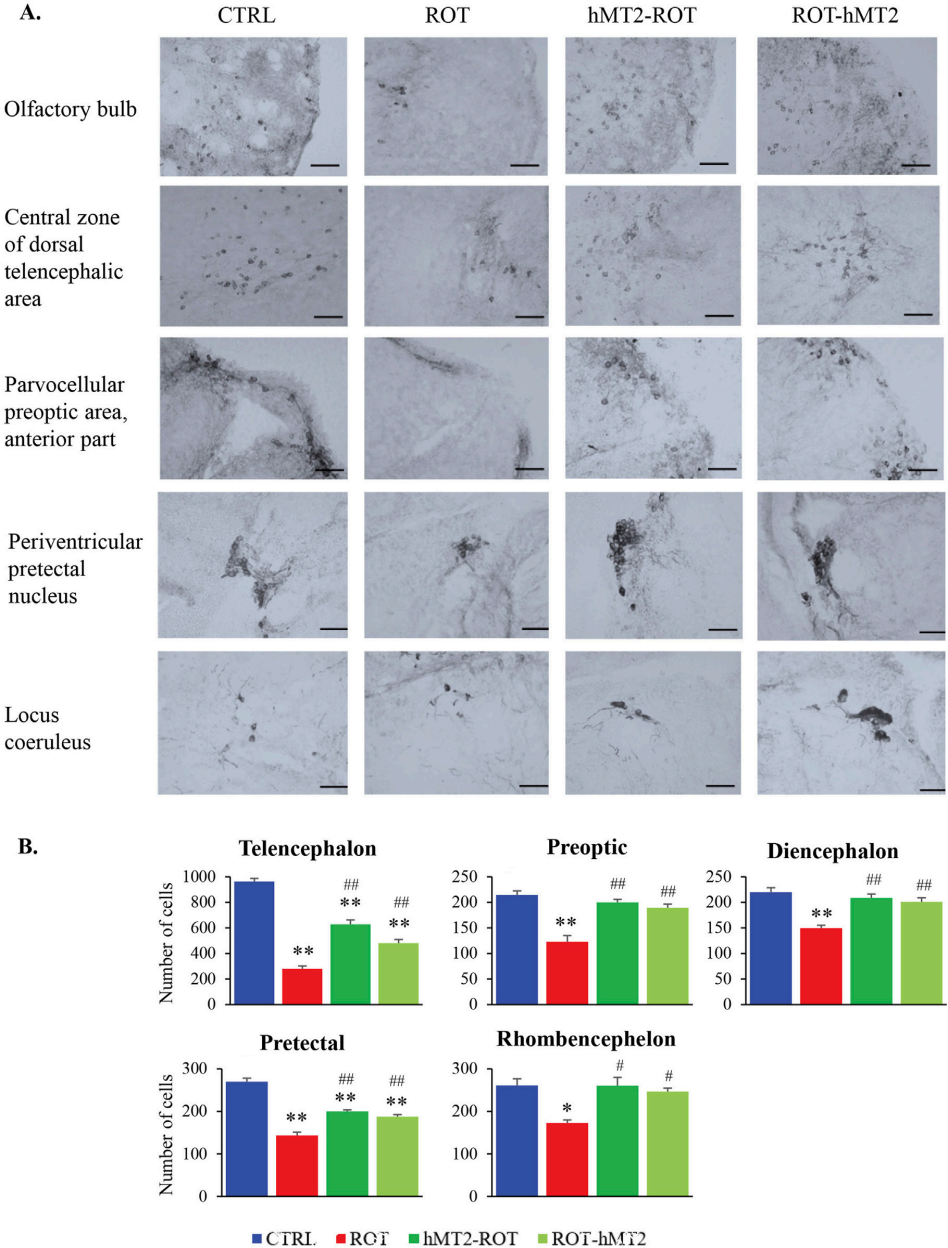
Effects of rotenone and hMT2 on dopaminergic neuron population. **(A)** Representative light micrographs of brain regions in CTRL, ROT, hMT2-ROT and ROT-hMT2 groups using tyrosine hydroxylase (TH) immunohistochemical staining. Scale bars = 50 μm. **(B)** Quantification dopaminergic neurons (TH + cells) within the zebrafish brain regions in CTRL, ROT, hMT2-ROT and ROT-hMT2 groups. *p < 0.05, **p < 0.001 vs. CTRL; #p < 0.05, ##p < 0.001 vs. ROT (n = 3 per group).

Dopaminergic neurons (TH^+^ cells) in the zebrafish brain are organized into distinct clusters or nuclei, across five regions: telencephalon, preoptic area, diencephalon, pretectal area and rhombencephalon. TH^+^ cells vary in size and shape, with smaller round cells located in the olfactory bulb, while larger cells with dendritic projections are observed in the locus ceruleus (Figure 6A).

In the ROT group, the number of TH^+^ cells showed a significant reduction in across all brain regions compared to the CTRL group (Figure 6B). The most substantial reduction was observed in the telencephalon (71%, p < 0.001), followed by the pretectal area (47%, p < 0.001), preoptic area (43%, p < 0.001), rhombencephalon (34%, p < 0.05) and diencephalon (32%, p < 0.001). Treatment with hMT2 attenuated the rotenone-induced loss of TH^+^ cells. The hMT2-ROT showed significantly higher numbers of TH^+^ cells in the telencephalon compared to ROT-hMT2 group (p < 0.05).

### Effects of rotenone and hMT2 on mitochondrial function

**FIGURE 7:**
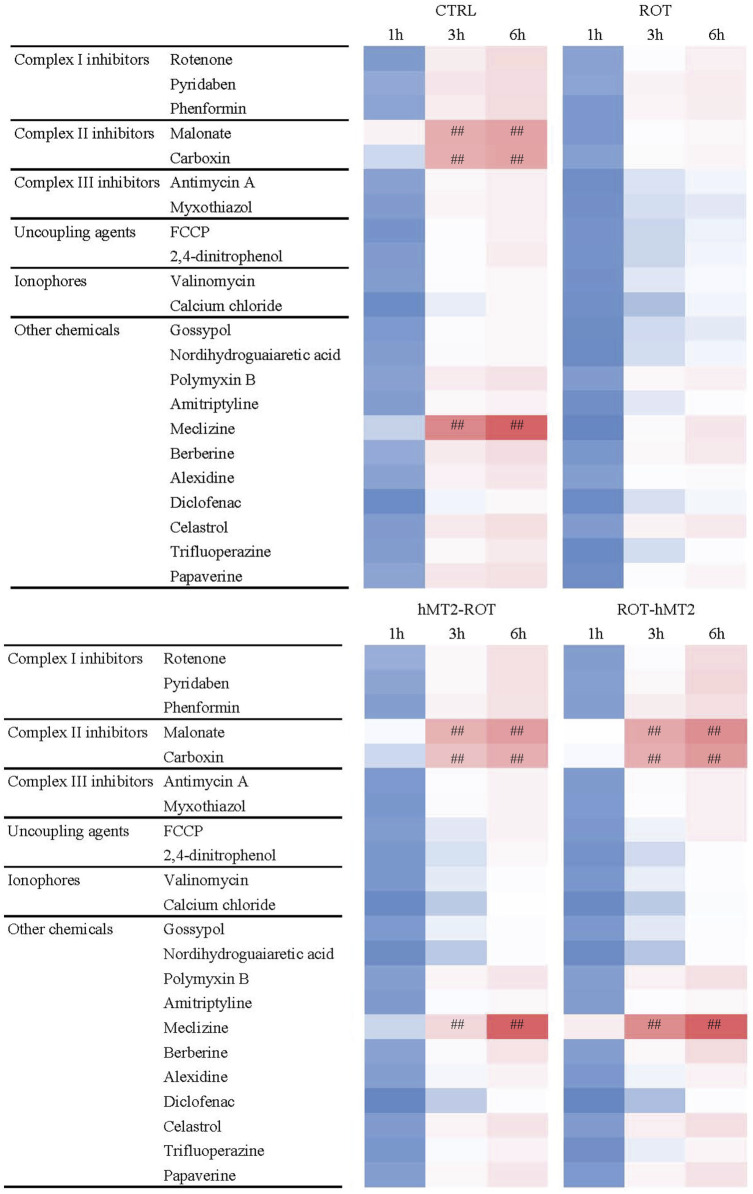
Effects of rotenone and hMT2 on mitochondria function. Representative phenetic maps of CTRL, ROT, hMT2-ROT and ROT-hMT2 mitochondria in the presence of mitochondria-centered drugs for 6 h, generated after normalization of the optical density values of each drug concentration at 590 nm (purple color) to those of the positive-control wells included in the MitoPlate™ I-1 plates. #p < 0.05, ##p < 0.001 vs. ROT (n = 3 per group).

**FIGURE 8:**
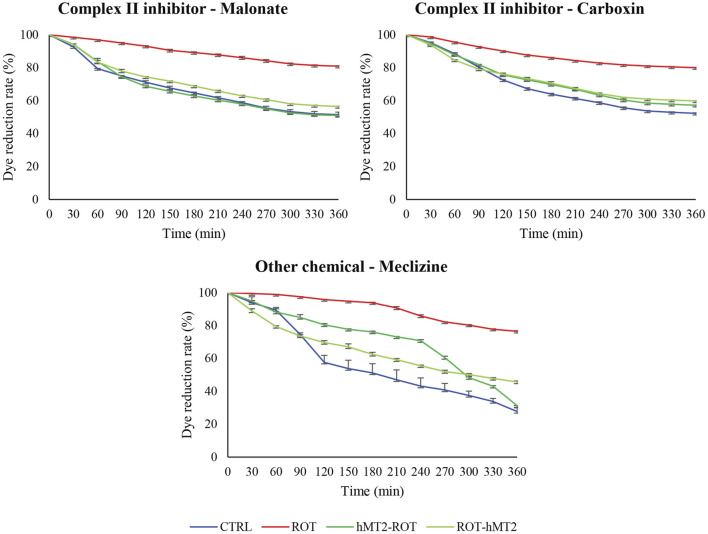
Effects of rotenone and hMT2 on dye reduction kinetics in mitochondria with complex II inhibitors (malonate and carboxin) and meclizine. The graphs showed representative reduction dynamics of the dye over time measured as absorbance at 590 nm for 6 h at 5-min intervals. ROT group exhibited significantly slower dye reduction, indicating mitochondrial dysfunction. Both hMT2-ROT and ROT-hMT2 groups showed improved dye reduction rates compared to ROT, suggesting restored mitochondrial function.

Lastly, we used the MitoPlate™ I-1 to measure mitochondrial sensitivity to 22 different mitochondrial inhibitors by measuring succinate oxidation rate, aiming to investigate whether hMT2 treatment affects the susceptibility of brain tissues to mitochondrial-centered inhibitors. The inhibitors comprise complex I, II and III inhibitors, uncoupling agents, ionophores, and other chemicals. The quantity and activity of mitochondrial machinery determine the extent of succinate oxidation in the samples. Greater inhibition of succinate oxidation by a specific inhibitor suggest a reduction in mitochondrial activity or decrease in the abundance of components involved in the succinate oxidation downstream processes. Additionally, varied responses to different inhibitors may indicate the presence or activity of particular metabolic pathways. A qualitative overview of the fold change results showed that the ROT group was significantly resistant to complex II inhibitors (malonate and carboxin), and meclizine, compared to the CTRL group (Figures 7, 8). hMT2 treatment in both hMT2-ROT and ROT-hMT2 groups reversed these effects.

## Discussion

### Effect of rotenone in adult zebrafish

Exposure to rotenone, a mitochondrial complex I inhibitor, successfully induced PD-like symptoms in zebrafish. These symptoms included impaired locomotor activity, decreased dopamine levels, increased oxidative stress, heightened inflammatory response, reduced dopaminergic neuron population and impaired mitochondrial function.

Rotenone-induced PD model demonstrated significant motor dysfunction, evidenced by reduced total swimming distance and increased anxiety-like behavior, as measured by the novel tank test (Figure 2). These results align with a previous study of motor deficits in rotenone-induced PD models (Wang et al., 2017). In contrast, Hettiarachchi et al. (2022) noted that while rotenone affects memory acquisition and consolidation in adult zebrafish, it did not induce anxiety in the latent learning maze test.

Following rotenone exposure, *mt2* and *smtb* expression was significantly upregulated (Figure 3A), which is in accordance with a previous study in MPTP-induced zebrafish PD model (Mohamad Najib et al., 2023). Additionally, a neuroprotective factor, *bdnf*, crucial for dopaminergic neuron survival, showed a significant reduction in *bdnf* expression after rotenone exposure (Figure 3B), consistent with previous findings (Bilge et al., 2020). BDNF binds to its primary receptor, tyrosine receptor kinase B (TrkB), activating the phosphatidylinositol 3-kinase/Akt (PI3K/Akt) signaling pathways to support neuronal survival. The downregulation of *bdnf* expression in PD is attributed to the aggregation of α-synuclein inhibiting TrkB production, which may lead to decreased activation of Akt, resulting in reduced *bdnf* expression (Singh et al., 2023).

Rotenone exposure also significantly altered the expression of several key genes involved in dopamine uptake and synthesis, namely, *dat*, *th1* and *th2* (Figure 4A). Our study found significant upregulation of *th1* and *th2* expressions in the zebrafish brain following rotenone exposure. In contrast, Wang et al. (2017) reported that TH expression was decreased by 50% in rotenone-treated zebrafish using Western blotting. Nevertheless, dopamine levels remained reduced in the brain of zebrafish treated with rotenone in our study (Figure 4B), which aligns with previous studies (Khotimah et al., 2015; Wang et al., 2017). We postulated that the increased expression of *th1* and *th2* may be due to a compensatory response to maintain dopamine synthesis to make up for the decreased dopamine levels in the brain caused by the loss of dopamine production capacity due to the death of dopaminergic neurons. Immunohistochemistry revealed a significant reduction in dopaminergic neurons in rotenone-treated zebrafish (Figure 6), correlating with observed motor deficits and lower dopamine levels, as seen in rodent models (Rocha et al., 2022).

Rotenone exposure induced a robust inflammatory response, indicated by elevated expression of pro-inflammatory cytokines (*il1a*, *il1b*, *tnfa* and *cox2*) (Figure 5A) and increased lipid peroxidation (Figure 5B). These findings are consistent with previous studies (Unal et al., 2020; Cansiz et al., 2021), which also documented a decrease in antioxidant enzymes (i.e., superoxide dismutase, glutathione S-transferase and glutathione) in the zebrafish brain following rotenone treatment.

Lastly, this study also investigated the effects of hMT2 on mitochondrial function, given that mitochondrial dysfunction is a hallmark of PD. Rotenone treatment caused a significant decrease in mitochondrial activity, as demonstrated by the significantly slower dye reduction rate in the presence of mitochondrial inhibitors: complex II inhibitors (malonate and carboxin) and meclizine (Figure 8). A previous study has reported that rotenone disrupts the maintenance of mitochondrial calcium balance (Yurtsever et al., 2020). Similarly, the ATP level and the activity of mitochondrial complex I were significantly decreased in rotenone-induced rodent model compared to control rat (Alikatte et al., 2021).

### Effects of hMT2 on rotenone-induced PD model

MT are known for neuroprotective effects in PD, such as reducing oxidative stress, inflammation and α-synuclein toxicity, all factors contributing to PD (Miyazaki and Asanuma, 2023). MT’s role in chelating metals such as copper and iron has been reported to inhibit α-synuclein aggregation and promote mitochondrial protection. The current study explored hMT2’s role in a rotenone-induced PD zebrafish model, which has not been extensively studied before. This study provides new insights into hMT2’s potential for restoring motor functions, dopamine levels, and mitochondrial function, reducing oxidative stress and inflammation, and rescuing dopaminergic neurons in the rotenone-induced zebrafish PD model.

Administration of hMT2, especially in the co-treatment group (ROT-hMT2), significantly improved locomotor activity (Figure 2A), suggesting that hMT2 can ameliorate rotenone-induced motor impairments. The improvements in motor function may be due to hMT2’s role in enhancing dopamine synthesis and mitigating dopaminergic neuron loss in the PD model. hMT2 treatment significantly downregulated the expression of *mt2* and *smtb* (Figure 3A), while up-regulating the expression of *bdnf* (Figure 3B), suggesting the neuroprotective effect of hMT2. Furthermore, the rotenone-induced dysregulated expressions of *dat*, *th1* and *th2* was also restored by hMT2 treatment (Figure 4A). Treatment with hMT2 notably increased the number of dopaminergic neurons (Figure 6B). This preservation of dopaminergic neurons is likely a key mechanism by which hMT2 exerts its neuroprotective effects. Additionally, the selective improvement in locomotor activity particularly in the ROT-hMT2 group suggested that hMT2 could enhance recovery processes rather than merely preventing rotenone-induced damage.

In PD, inflammation and oxidative stress play crucial roles in dopaminergic neuron degeneration (Marogianni et al., 2020). Thus, the reduction in oxidative stress is particularly important in PD. hMT2 administration significantly reduced cytokine expression and lipid peroxidation, highlighting its anti-inflammatory and antioxidant properties, which are well known characteristics of MT. Similarly, previous study in rat spinal cord injury model has shown that exogenous MT adiminstration reduced reactive oxygen species (ROS) production and lipid peroxidation (Rios et al., 2018). Furthermore, MT2A overexpression in bladder cancer cell lines significantly downregulated endogenous ROS production and alleviated H_2_O_2_-induced ROS, further confirming MT’s antioxidative properties (Sung et al., 2022). Additionally, our study showed that hMT2 treatment improved dye reduction rates, indicating its role in mitigating rotenone-induced mitochondrial impairments, particularly in the presence of mitochondrial complex II inhibitors (malonate and carboxin) and meclizine (Figure 8). The effect of hMT2 on complex II inhibitors might be related to its role in maintaining succinate oxidation, specifically affected by rotenone-induced dysfunction. However, the role of MTs in the primary mitochondrial function is not well supported by experimental data. Nevertheless, previous study reported that MT1A tranduced into the mitochondria of MPP^+^-treated SH-SY5Y neuroblastoma cells was able to alleviate mitochondrial damage, as shown by restored ATP levels, mitochondrial NADH dehydrogenase activity and mitochondrial superoxide levels (Kang et al., 2018).

MT is predominantly produced by astrocytes in the brain and is secreted extracellularly (Saenz-Antonanzas et al., 2024), and the secreted MTs have been shown to protect dopaminergic neurons against 6-OHDA toxicity (Isooka et al., 2020). Administered hMT2 acts extracellularly, exhibiting neuroprotective effects through antioxidant and anti-inflammatory properties. However, astrocyte-derived MTs may be internalized into neurons via interaction with surface receptors belonging to the family of low-density lipoprotein receptor (LDL-R)-related proteins (LRP), such as LRP-1 and LRP-2 (also known as megalin). Jakovac et al. (2018) reported that megalin-positive neurons showed MT immunoreactivity in their membranes and within the cytoplasm, which in turn activated the signal transduction pathways, such as AKT1/Protein kinase B, supporting neuronal survival. In view of this, administration of hMT2 may perform neuroprotective actions extracellularly and inhibit intraneuronal pathogenic pathways.

While MT’s neuroprotective effects are well documented, this study contributes further insights into the specific mechanisms elicited by hMT2, such as its influence on dopamine-related gene expression (*dat*, *th1* and *th2*), and the mitochondrial protection offered by hMT2.

It is crucial to recognize some limitations that might have affected the results, even though this study provides insightful information about the neuroprotective effects of hMT2 in the rotenone-induced zebrafish PD model. A vehicle control group was not included in this study because the effects of rotenone in zebrafish are well-documented in the literature, and the focus was on assessing the neuroprotective effects of hMT2. Nevertheless, we acknowledge the importance of including a vehicle group to rule out the possibility of buffer-induced effects.

## Conclusion

This study highlights the neuroprotective potential of MT, particularly hMT2, in a rotenone-induced PD model in adult zebrafish. hMT2 treatment alleviates motor deficits, restores dopamine levels, reduces inflammation and oxidative stress, preserves dopaminergic neurons, and improves mitochondrial function. These findings support the potential of MT as a therapeutic target for PD, warranting further investigation into its mechanisms of action and efficacy in clinical settings, while expanding on previous findings by validating its effects in different models and conditions.

## Data Availability

The raw data supporting the conclusions of this article will be made available by the authors, without undue reservation.
